# Ethanol Induces Enhanced Vascularization Bioactivity of Endothelial Cell-Derived Extracellular Vesicles via Regulation of MicroRNAs and Long Non-Coding RNAs

**DOI:** 10.1038/s41598-017-14356-2

**Published:** 2017-10-23

**Authors:** Tek N. Lamichhane, Christopher A. Leung, Lampouguin Yenkoidiok Douti, Steven M. Jay

**Affiliations:** 1 Fischell Department of Bioengineering, University of Maryland, College Park, MD 20742 United States; 2 Marlene and Stewart Greenebaum Comprehensive Cancer Center, University of Maryland Medical Center, Balitmore, MD 21201 USA; 3 Program in Molecular and Cell Biology, University of Maryland, College Park, MD 20742 USA

## Abstract

Extracellular vesicles (EVs), such as exosomes, have been identified as regulators of vascular remodeling and have promise as therapeutics for vascularization applications. Towards development of EVs as therapeutics, it has been demonstrated that physiological stimuli of angiogenic phenotypes in EV-producing cells can enhance the potency of EVs for vascularization. The goal of this study was to assess whether ethanol, which induces angiogenic phenotypes in endothelial cells, could be employed to enhance endothelial-derived EV vascularization bioactivity. The results indicate that ethanol conditioning of endothelial cells increases the ability of endothelial EVs to induce a pro-vascularization response. This response is due in part to increased CD34 expression in recipient endothelial cells that may result from downregulation of microRNA-106b in EVs isolated from ethanol-conditioned producer endothelial cells. Further, ethanol-induced upregulation of long non-coding RNAs (lncRNAs) HOTAIR and MALAT1 in endothelial EVs was observed to play a significant role in mediating pro-angiogenic effects of these vesicles. Overall, these studies validate ethanol conditioning as a method to enhance the bioactivity of endothelial EVs via regulation of EV-associated microRNAs (miRNAs) and, especially, lncRNAs. Further, the results suggest that alcohol consumption may activate endothelial EVs towards a pro-vascularization phenotype, which could have implications for alcohol-induced tumor angiogenesis.

## INTRODUCTION

Extracellular vesicles (EVs), including exosomes, microvesicles and other subtypes of cell-derived vesicles, have emerged as both critical mediators of intercellular communication as well as potential therapeutic vectors for a variety of applications^1–3^. Among the myriad applications and physiological systems in which EVs have been explored, their roles in angiogenesis and vascular remodeling are some of the most consistently reported. The physiological relevance of EVs in mediating vascular cell-cell communication and remodeling has been established^4–8^, and EVs have been applied for therapeutic vascularization in a number of settings^9–17^. This use of EVs as therapeutics for vascularization is especially intriguing, as EVs may offer a combination of properties that could overcome some limitations associated with conventional cell-based and molecular therapeutics for this application. Specifically, compared to molecules, EVs are multifactorial vectors capable of stimulating multiple signaling and gene regulation pathways, while compared to cells, EVs have defined half-lives and clearance pathways and are not capable of uncontrolled division or differentiation. Further, EVs have been shown to mediate the paracrine pro-angiogenic effects of cells^18^.

However, EVs also have limitations as therapeutic vectors. Specifically, they may have low potency due to low microRNA (miRNA) content per vesicle^19^, given that miRNAs have been identified as crucial components mediating vascularization bioactivity of EVs^14,16,17^. Methods to enhance the potency of EVs have been developed, including exogenous loading approaches^20–26^ and cell conditioning via exposure to hypoxia or growth factor stimulation^17,27,28^. However, these approaches may not be easily adaptable to large-scale biomanufacturing of therapeutic EVs for vascularization applications, thus limiting translational potential.

One substance that may be straightforwardly incorporated into scalable EV production that also induces a pro-vascularization phenotype in endothelial cells is ethanol^29–31^. Ethanol is already part of large-scale biotechnology production schemes and is relatively cheap and readily available compared to purified growth factors. Ethanol has been shown to induce angiogenic endothelial phenotypes via a variety of pathways^31–34^ and has also been shown to influence the bioactivity and cargo of EVs in other cellular systems^35,36^. However, there are scarce reports of how ethanol effects on endothelial cells impact bioactivity of EVs derived from these cells.

We hypothesized that ethanol conditioning might increase the vascularization bioactivity of endothelial cell-derived EVs. In these studies, we sought to determine how cellular changes in endothelial cells induced by ethanol are manifested in EVs and to identify specific mechanisms of ethanol-induced regulation of endothelial cell EV activity. We report that ethanol increases the vascularization bioactivity of endothelial cell EVs through at least two distinct mechanisms: downregulation of anti-angiogenic miRNA cargo (miR-106b) and upregulation of pro-angiogenic long non-coding RNA (lncRNA) cargo (MALAT1 and HOTAIR). These findings have implications for generation of EVs for therapeutic vascularization applications and also may shed light on the role of EVs in alcohol-induced angiogenesis in cancer and other physiological settings.

## Results

### Ethanol stimulates EV production by endothelial cells

**Figure 1:**
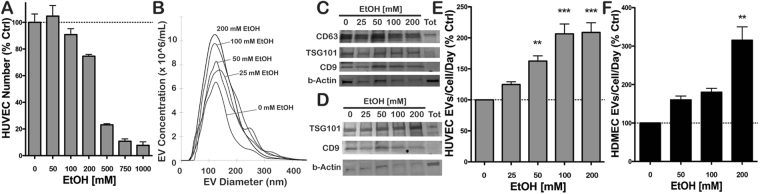
Endothelial cell production of EVs in the presence of alcohol. (**A**) HUVEC survival was assessed with the indicated concentrations of ethanol (EtOH) included in growth medium (EGM2) (n = 3). (**B**) HUVEC EV size distribution in the presence or absence of the indicated concentrations of EtOH in the medium was assessed via NanoTracking Analysis (NTA) using a NanoSight LM10 (n = 3). Immunoblots for proteins associated with exosomes were performed for (**C**) HUVEC EVs and (**D**) HDMEC EVs isolated from producer cells cultured with the indicated EtOH concentrations. Blots shown are representative of three independent experiments. EV production rate by cells in medium containing the indicated EtOH concentrations was determined by NTA for (**E**) HUVEC and (**F**) HDMEC; n = 3, **P < 0.01, ***P < 0.001 compared to 0 mM EtOH condition.

As a first step in evaluating the potential of ethanol conditioning as a means to enhance vascularization bioactivity of endothelial cell-derived EVs, the effects of ethanol on EV production were investigated. Concentrations of ethanol beyond 100 mM were found to induce significant cell toxicity in human umbilical vein endothelial cells (HUVECs) (Fig. 1A), thus 100 mM was used as a maximum ethanol level in most experiments. The inclusion of ethanol in the culture medium did not appear to affect the structural integrity of produced EVs, as mean diameters (Fig. 1B) and protein expression levels (Fig. 1C,D) were found to be similar over the range between 0-200 mM ethanol for both HUVEC and human dermal microvascular endothelial cell (HDMEC) EVs (representative blots shown in Supplementary Fig. S1). Notably, up to ~2–3 fold increased EV production by endothelial cells was observed at higher ethanol concentrations (Fig. 1E,F).

### Ethanol conditioning increases endothelial cell-derived EV vascularization bioactivity

**Figure 2:**
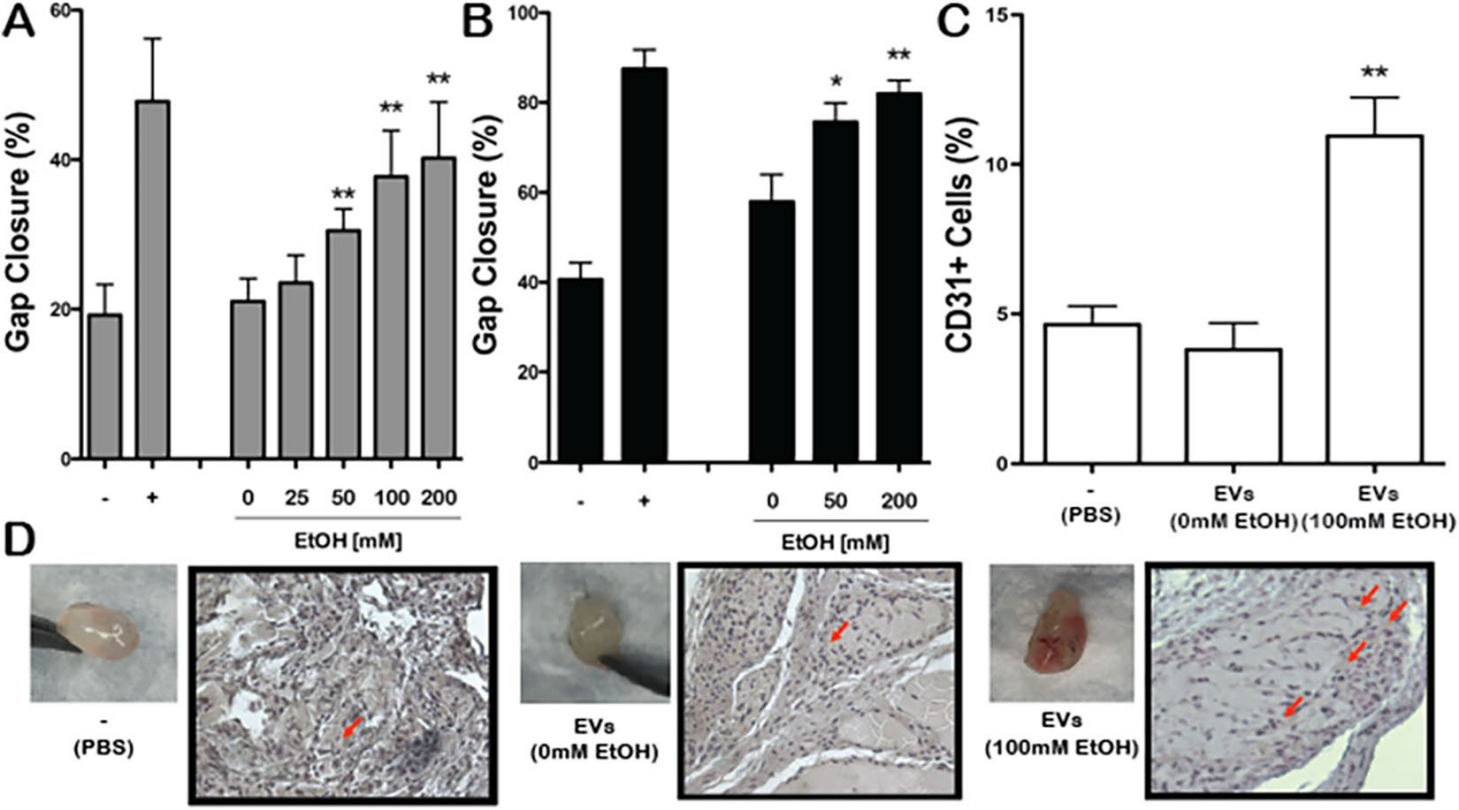
Ethanol conditioning increases endothelial cell-derived EV vascularization bioactivity. Gap closure by (**A**) HUVECs and (**B**) HDMECs was assessed following stimulation by 100 µg/ml EVs isolated from the same producer cell type in medium with the indicated ethanol (EtOH) concentrations (n = 3, *P < 0.05, **P < 0.01 vs. 0 mM EtOH condition); HUVECs incubated in basal medium (EBM2, without growth factors) were used as the negative control (−) and HUVECs incubated in growth medium (EGM2, with growth factors) were used as positive controls (+). (**C**) Matrigel plugs injected into C57Bl/6 mice containing PBS as a negative control (−) or 100 µg HUVEC EVs from cells cultured with the indicated concentrations of EtOH in the media were removed 10 d after implantation and CD31+ cells were quantified using immunohistochemical staining (n = 6; **P < 0.01 compared to all other groups by one-way ANOVA). Data are presented as %CD31+ stained cells out of all cells counted for the gel sections from a given animal. (**D**) Representative gel images and immunohistochemistry sections from animals in the indicated groups are shown (n = 6).

Following from the original hypothesis and given that structurally intact EVs were produced by endothelial cells in the presence of ethanol, EV vascularization bioactivity was directly assessed *in vitro* using an endothelial cell gap closure assay and *in vivo* using a Matrigel plug injection mouse model. EVs isolated from both HUVECs (Fig. 2A) and HDMECs (Fig. 2B) showed increased stimulation of endothelial gap closure based on the ethanol concentration used to condition the EV-producing cells. Additionally, ethanol conditioning (100 mM) endowed endothelial cell-derived EVs with increased ability to recruit host CD31+ cells into injected Matrigel plugs in C57Bl/6 mice (Fig. 2C,D). Together, these data demonstrate increased vascularization bioactivity of EVs derived from endothelial cells cultured in the presence of ethanol compared to those isolated from endothelial cells without ethanol conditioning.

**Figure 3:**
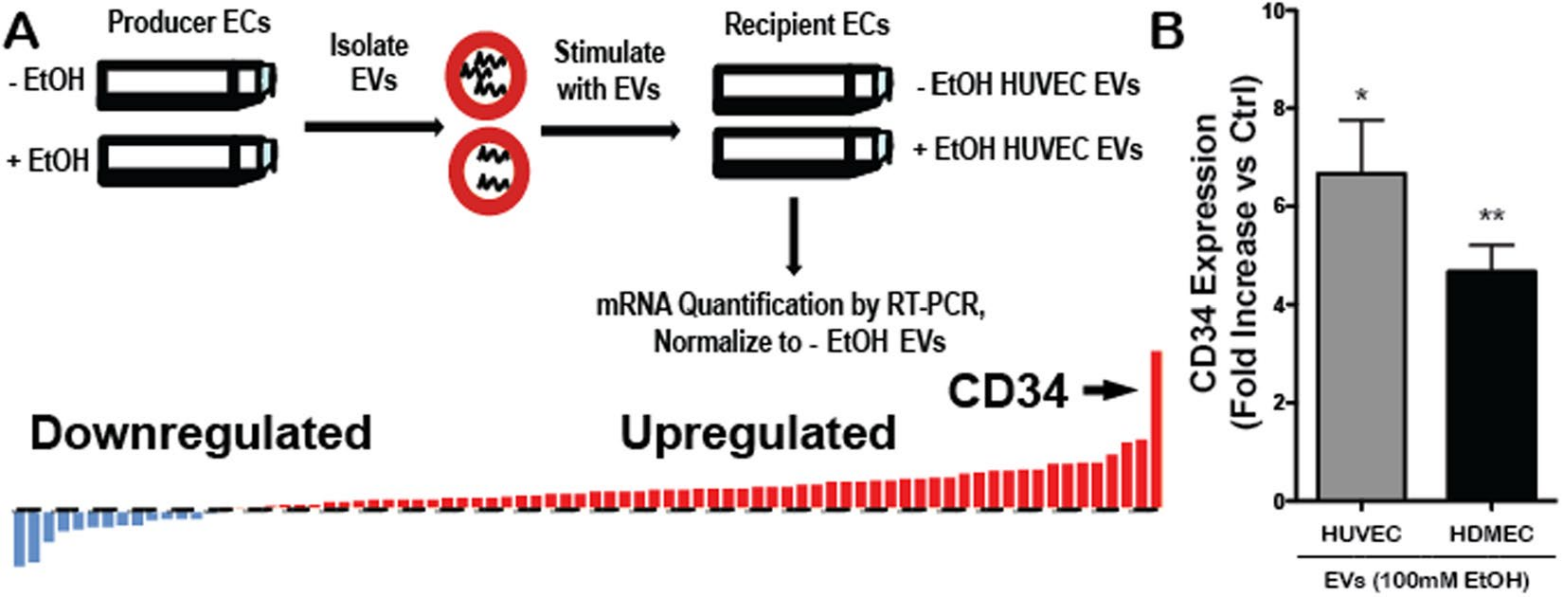
CD34 is upregulated in endothelial cells (ECs) stimulated by EVs from ethanol (EtOH)-conditioned ECs. (**A**) Experimental schematic and representation of gene regulation profile of 92 genes associated with angiogenesis. Data shown represent mRNA levels in recipient HUVECs stimulated by 100 µg/ml EVs from producer HUVECs cultured in the presence vs. the absence of 100 mM EtOH for 24 h. (**B**) CD34 expression in recipient HUVECs and HDMECs following stimulation by 100 µg/ml EVs from producer ECs of the same type cultured in the presence or absence (control (Ctrl)) of 100 mM EtOH for 24 h was assessed by qPCR using distinct primers from those in the mRNA array (n = 3; *P < 0.05, **P < 0.01).

To determine the specific mechanism of the enhanced vascularization bioactivity of endothelial cell-derived EVs induced by ethanol conditioning of producer endothelial cells, we first profiled gene expression in recipient endothelial cells upon stimulation with EVs isolated from producer endothelial cells cultured in the presence or absence of 100 mM ethanol. Among 92 genes examined that are associated with angiogenic activity, a majority (68 of 92) were upregulated upon stimulation by EVs from ethanol-conditioned HUVECs compared to unconditioned HUVEC-derived EVs (Fig. 3A, Supplementary Table S1). The mRNA of CD34, a sialomucin protein associated with endothelial cell migration and angiogenesis^37^, was the most significantly differentially regulated (upregulated ~5 fold). This finding was confirmed independently by qPCR using different primers in both HUVECs and HDMECs (Fig. 3B).

### Ethanol conditioning regulates endothelial cell-derived EV miRNA content

**Figure 4:**
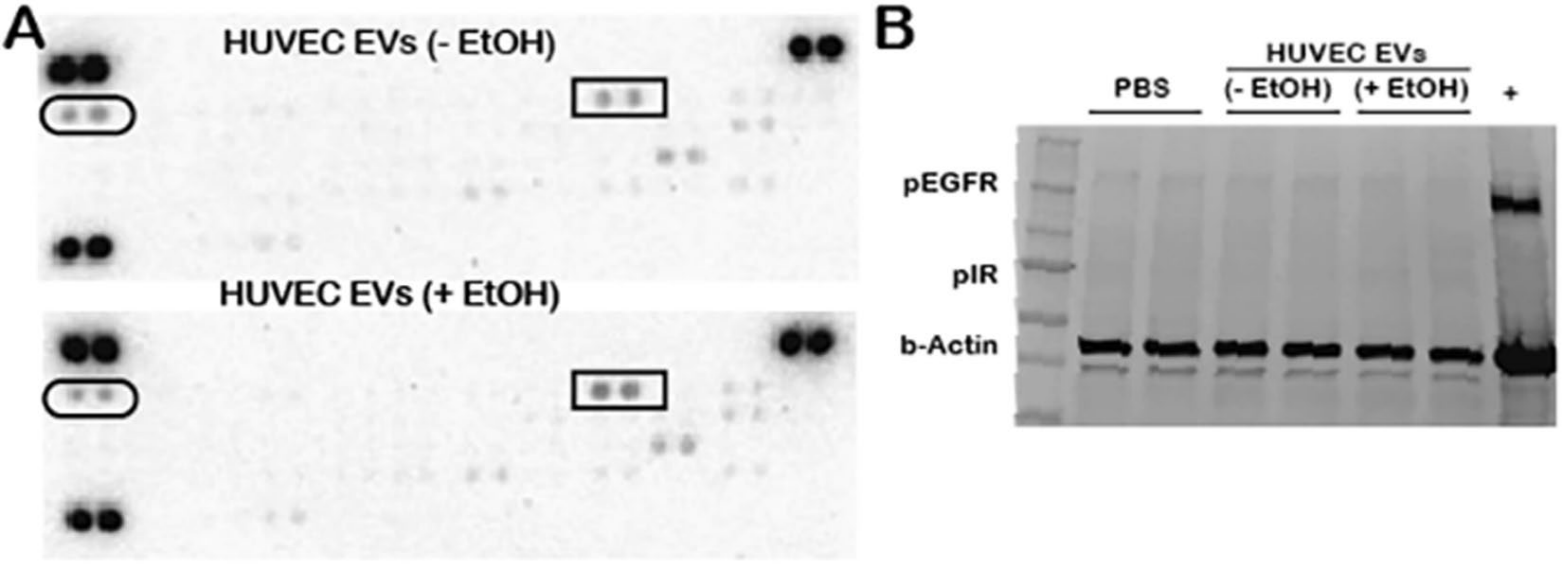
Endothelial cell receptor tyrosine kinase stimulation by EVs. (**A**) Phosphorylated RTK arrays were incubated for 30 min with lysates from recipient HUVECs stimulated by 100 µg/ml EVs from producer HUVECs cultured in the presence or absence of 100 mM ethanol (EtOH) for 24 h. Ovals indicate phospho-epidermal growth factor receptor (pEGFR) and rectangles indicate phosphor-insulin receptor (pIR). Blots shown are representative of three independent experiments. (**B**) The same conditions were used to generate separate immunoblots for pEGFR and pIR using different antibodies (n = 3; H1975 cells were used as a positive control for pEGFR (+)).

To determine the specific cause of the measured CD34 upregulation, EV-associated protein signaling and miRNA-mediated information transfer were examined, as proteins can be integrated within EV membranes to induce receptor-mediated signal transduction while miRNAs are among the most prevalent and biologically significant components of EV cargo^1,3^. Assessment of phosphorylation of 49 common receptor tyrosine kinases (RTKs) (Supplementary Table S2) that might be activated by ectodomains of EV-associated proteins did not reveal any significant differences between groups (Fig. 4A). This finding was partially confirmed by independent immunoblots for the phosphorylated versions of two RTKs that were stimulated to the greatest degree in this experiment, epidermal growth factor receptor (pEGFR) and insulin receptor (pIR) (Fig. 4B).

**Figure 5:**
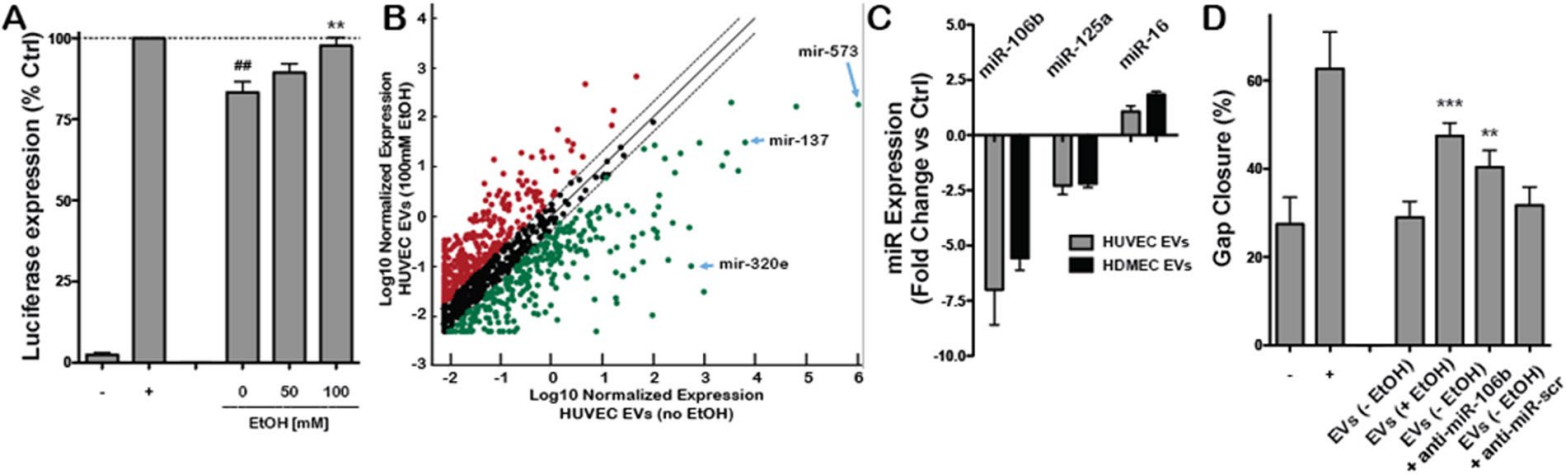
Ethanol (EtOH) regulates endothelial cell-derived EV microRNA content. (**A**) Luciferase expression in recipient HUVECs from a construct containing the 3′ untranslated region of CD34 was measured by bioluminescence imaging following stimulation by 100 µg/ml EVs from producer HUVECs cultured in the presence of the indicated concentrations of EtOH for 24 h (n = 4; **P < 0.01 vs. 0 mM EtOH condition, ^##^P < 0.01 vs. positive control (+) condition). Mock-transfected HUVEC not exposed to EVs were used as negative controls (−), while transfected HUVEC not exposed to EVs were used at the positive control (+). (**B**) Average whole miRNome array results comparing miR content of EVs derived from HUVEC cultured in the presence or absence of 100 mM EtOH for 24 h (n = 3). Red dots indicate upregulation of miRs and green dots indicate downregulation of miRs in HUVEC EVs based on producer cell EtOH exposure. Black dots indicate similar expression levels. (**C**) Expression levels of the indicated microRNAs (miRs) in endothelial cell EVs from producer cells cultured in the presence vs. absence of 100 mM EtOH for 24 h was determined by qPCR (n = 3). (**D**) Gap closure of HUVECs was assessed upon stimulated by EVs derived from HUVECs cultured in the absence (−EtOH) or presence (+EtOH) of 100 mM EtOH for 24 h following mock transfection or transfection by an antagomir to miR-106b (anti-miR-106b) or a scrambled oligo sequence (anti-miR-scr) (n = 3; ***P < 0.001, **P < 0.01 vs. EVs (−EtOH) group). HUVECs incubated in basal medium (EBM2, without growth factors) were used as the negative control (−) and HUVECs incubated in growth medium (EGM2, with growth factors) were used as positive controls (+).

Potential EV-mediated miRNA regulation of CD34 expression was assessed using a luciferase reporter assay, where the 3′-untranslated region (UTR) of CD34 was cloned into a luciferase vector that was subsequently transfected into HUVECs. EVs from HUVECs cultured in the presence or absence of 100 mM ethanol were then applied to recipient HUVECs, and detection of miRNA-specific targeting of CD34 for downregulation was performed based on quantification of luciferase expression. The results of this assay indicated that EVs from non-conditioned producer HUVECs contain miRNAs that specifically target CD34 for downregulation in recipient cells, as these EVs induced a 16.7+/−3.3% reduction in luciferase activity compared to control (Fig. 5A). However, EVs from HUVECs cultured in the presence of 100 mM ethanol induced only a 2.3+/−2.5% reduction in luciferase activity (Fig. 5A), suggesting that ethanol conditioning of HUVECs alters EV miRNA content resulting in reduced CD34 degradation.

Towards identifying specific miRNA participants in this effect, analysis of the whole miRNomes of EV populations isolated from HUVECs cultured in the presence or absence of 100 mM ethanol was conducted. These studies revealed significant differences in miRNA content between the EV groups (Fig. 5B, Supplementary Table S3), including substantial downregulation of several miRNAs that are reported to negatively regulate angiogenesis, such as miR-573^38^, miR-137^39^, miR-320e^40^, and others. Further investigation of miRNAs that have been validated to target CD34 for downregulation, including miR-106b^41^, miR-125a^42^ and miR-9^42^, showed that miR-106b was downregulated > 5 fold in EVs from both HUVECs and HDMECs cultured in the presence of 100 mM ethanol compared to cells cultured without ethanol (Fig. 5C). To confirm the specific role of miR-106b, HUVECs (in the absence of ethanol) were transfected with an antagomir that was validated to significantly decrease miR-106b expression in HUVEC-derived EVs (Supplementary Fig. S2), recapitulating a specific effect of ethanol conditioning. Assessment of the vascularization bioactivity of these EVs, derived from non-ethanol-conditioned HUVECs but with diminished levels of miR-106b, showed increased ability to induce endothelial gap closure compared to control non-conditioned HUVEC EVs as well as HUVEC EVs from cells transfected with a scrambled antagomir sequence (Fig. 5D). Thus, specific downregulation of miR-106b partially recapitulated a significant phenotypic effect of ethanol conditioning on endothelial cell-derived EVs. Overall, these data point to regulation of miR-106b as a crucial component of the mechanism of enhancement of endothelial cell-derived EV vascularization bioactivity by ethanol conditioning.

### Ethanol conditioning regulates endothelial cell-derived EV long non-coding RNA content

**Figure 6:**
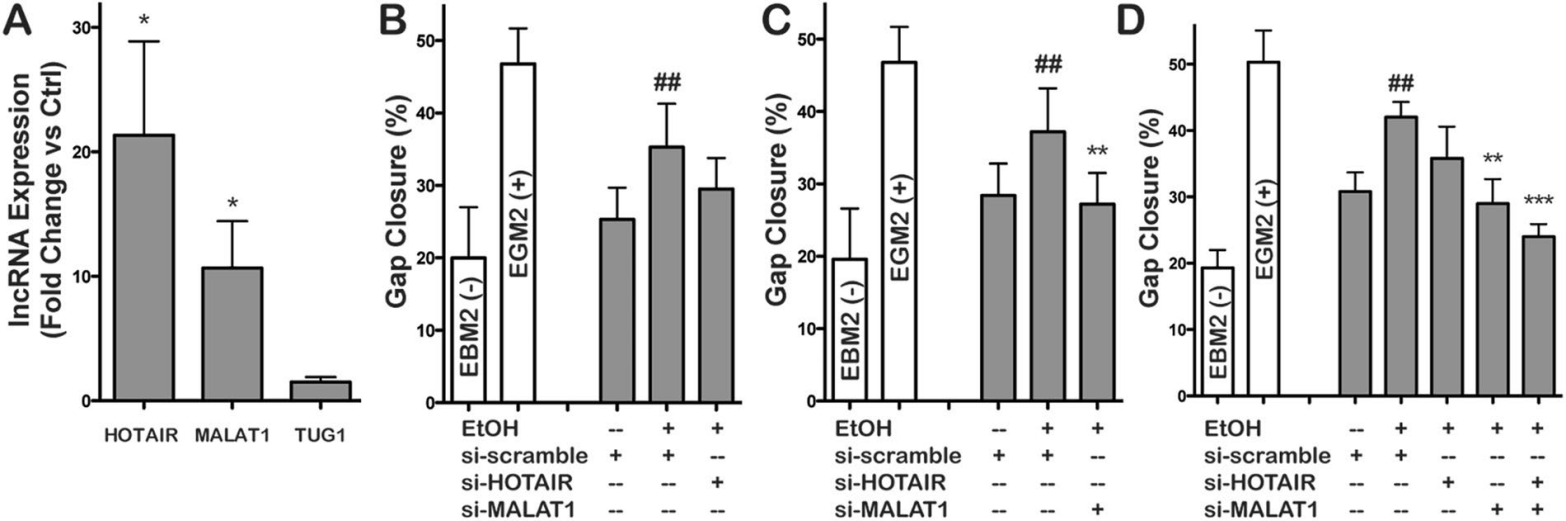
Ethanol regulates endothelial cell-derived EV lncRNA content. (**A**) Expression levels of the indicated lncRNAs were assessed by qPCR in EVs from HUVECs cultured in the presence vs. absence of 100 mM EtOH for 24 h (n = 3; *P < 0.05). (**B**–**D**) HUVEC gap closure was assessed following 24 h stimulation by 100 µg/ml EVs from HUVECs cultured in the absence (−EtOH) or presence (+EtOH) of 100 mM EtOH for 24 h following transfection with a scrambled siRNA (scr) or siRNA specific to (B) HOTAIR, (**C**) MALAT1, or (**D**) both HOTAIR and MALAT1 (double transfection) (n = 4; ^##^P < 0.01 vs. – EtOH+ scr; **P < 0.01, ***P < 0.001 vs. +EtOH+ scr). HUVECs incubated in basal medium (EBM2, without growth factors) were used as the negative control (−) and HUVECs incubated in growth medium (EGM2, with growth factors) were used as positive controls (+).

The ethanol-induced downregulation of miRNAs that negatively regulate angiogenesis is not necessarily sufficient to induce pro-vascularization bioactivity in endothelial cell EVs. To assess additional possible molecular players, long non-coding RNA (lncRNA) content in EVs was investigated, as lncRNAs have been reported as EV cargo components^43^. Specifically, lncRNAs known to induce angiogenesis via regulation of endothelial cell function, including HOTAIR^44^, MALAT1^45^, and TUG1^45,46^ were assessed by qPCR. Both HOTAIR and MALAT1 were significantly upregulated in EVs from HUVECs cultured in the presence of 100 mM ethanol (Fig. 6A). To assess specific roles of these lncRNAs, an approach similar to that described for miR-106b was used. In this case, HUVECs were transfected with siRNA specific to either HOTAIR (Fig. 6B), MALAT1 (Fig. 6C), both HOTAIR and MALAT1 (double transfection, Fig. 6D) or a scrambled siRNA sequence. The depletion of HOTAIR and MALAT1 from cellular RNA and EV RNA was confirmed by q-PCR (Supplementary Figs S3 and S4). The cells were then cultured in the presence or absence of 100 mM ethanol and assessment of the vascularization bioactivity of the EVs isolated from these groups of cells was conducted using endothelial gap closure assays. These experiments revealed that downregulating MALAT1 expression alone and MALAT1 and HOTAIR expression together significantly abrogated the increase in vascularization bioactivity in HUVEC EVs associated with ethanol conditioning (Fig. 6C,D). Overall, these data indicate that lncRNA regulation is a crucial aspect of ethanol-induced effects on endothelial cell EV bioactivity.

## Discussion

This study establishes, for the first time, that ethanol induces enhanced vascularization bioactivity in endothelial cell-derived EVs. Regulation of both EV-associated miRNAs and lncRNAs by ethanol conditioning of producer cells contributes to this effect, which has potential ramifications for therapeutic EV biomanufacturing. These data also suggest that EVs may play an important role in the mechanism of alcohol-induced angiogenesis in cancer.

The finding that miRNAs were regulated in this study is not surprising, as miRNA content has been consistently linked to EV vascularization bioactivity^14,16,17^ and ethanol exposure has been specifically linked to regulation of EV miRNAs^35,36^. However, the upregulation of CD34 expression in recipient endothelial cells resulting from ethanol-induced regulation of endothelial cell EV miRNA cargo is interesting and suggests that ethanol exposure may induce transformation of quiescent endothelial cells into angiogenic tip cells via EVs^37^. Also unexpected was the finding that ethanol conditioning impacts endothelial cell EV bioactivity via regulation of lncRNA cargo, specifically HOTAIR and MALAT1. LncRNAs are reported to constitute a much smaller proportion of total nucleic acid cargo of EVs when compared to miRNAs^43^. Yet, our results establish that these molecules may play significant roles in defining EV vascularization bioactivity, supporting other studies that have identified bioactive lncRNAs in EVs^47,48^. Ethanol-induced regulation of pro-angiogenic lncRNA is not unprecedented; MALAT1 has been found to be upregulated in brains of human alcoholics^49^. However, to our knowledge, this is the first report connecting EV-associated MALAT1 with angiogenic bioactivity. Thus, more extensive study of the effects of ethanol on lncRNA expression in endothelial cells and their EVs may be of interest. Further, therapeutic loading of lncRNAs such as MALAT1 into EVs may be a promising strategy for enhancing vascularization bioactivity.

**Figure 7:**
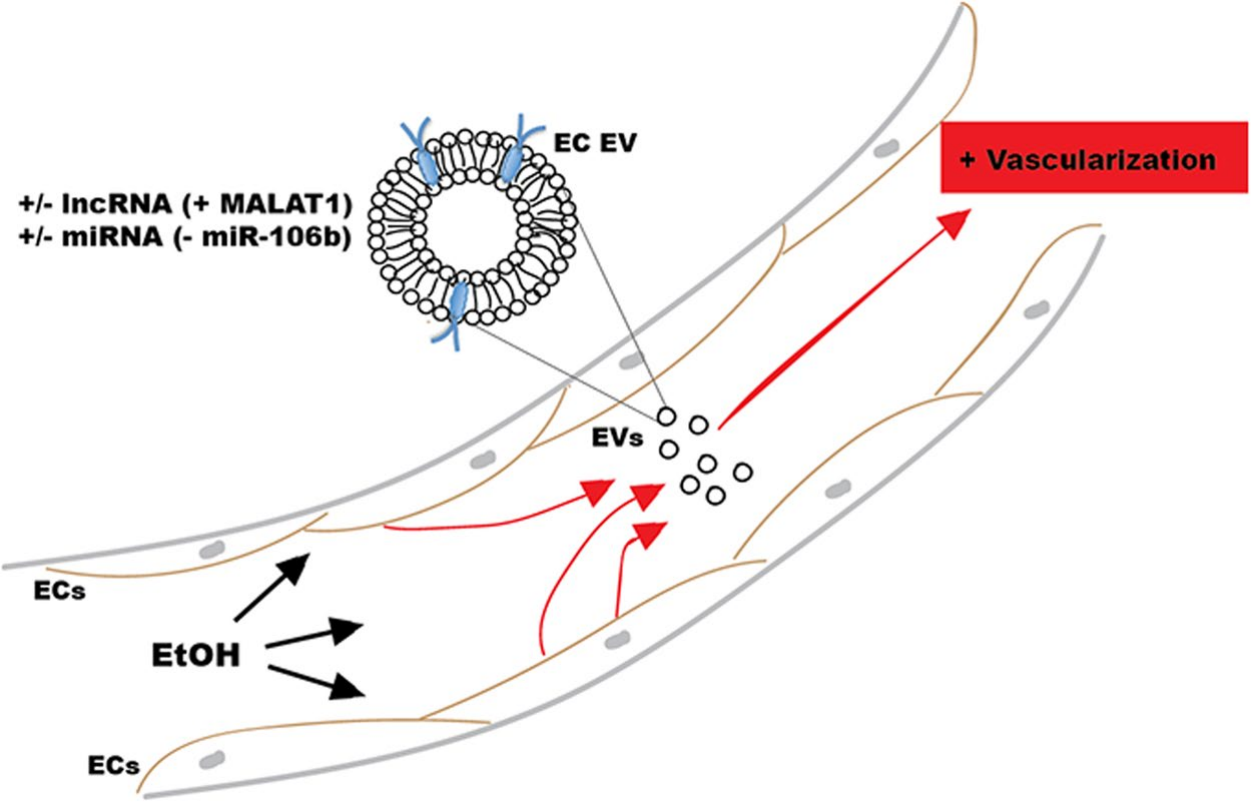
Schematic of potential mechanism of alcohol effects on endothelial cell (EC) EVs. EtOH = ethanol, miRNA = microRNA, lncRNA = long non-coding RNA.

Finally, as previously stated, the finding that ethanol increases the angiogenic bioactivity of endothelial cell EVs has important implications in cancer. Alcohol consumption has been identified as a risk factor for several cancers, with the links between alcohol and breast and liver cancers being particularly strong. For example, epidemiological studies have consistently shown that breast cancer risk increases with alcohol intake^50–53^. Among many potential mechanisms, alcohol-induced tumor angiogenesis has been identified as a possible driver of breast cancer progression^33,34^, with several signaling pathways in endothelial cells being implicated^30,31^. While activation of these pathways is typically associated with proteins, this study suggests that alcohol exposure to endothelial cells could induce production of pro-angiogenic EVs, which could potentially promote progression of latent tumors (Fig. 7). This possibility is further supported by reports that EVs have been shown to stimulate tumor angiogenesis^7,9,10,18,54,55^. Thus, the results of this study support further exploration of the potential mechanistic role of endothelial cell-derived EV regulation in alcohol-induced cancer progression.

In summary, this study demonstrates that the pro-angiogenic effects of ethanol on endothelial cells increase the vascularization bioactivity of EVs derived from these cells. This is accomplished in part by regulation of both miRNA and lncRNA components of EVs, with miR-106b downregulation and MALAT1 upregulation having significant effects. These findings could inform future mechanistic studies of the role of EVs in alcohol-induced cancer progression and may also identify new therapeutic cargo or quality control parameters for large-scale biomanufacturing of EVs for therapeutic vascularization applications.

## Methods

### Cell culture

HUVEC and HDMEC were obtained from Promocell and were generally cultured in EGM2 medium (with growth factors) (Lonza). In gap closure experiments, EBM2 media (without growth factors) (Lonza) was used as the basal medium. All media were filter sterilized and depleted of serum-derived EVs prior to use by centrifuging complete media at 100,000 × g for 16 h at 4 °C. For all experiments involving ethanol conditioning, cells were incubated in medium containing ethanol at the indicated concentration for 24 h.

### EV isolation and characterization

To isolate EVs, ~2 × 10^6^ cells were seeded into T175 flasks and allowed to incubate for 24 h or until ~50% confluent. Medium was then aspirated, cells were washed once with 20 ml of pre-warmed 1X PBS, and 35 ml of pre-warmed EV-depleted medium with or without ethanol was added to the flask. Cells were incubated until ~90% confluent, medium was collected and differential centrifugation was employed to isolate EVs as previously described^20^. The concentration and size of vesicles were measured by NanoTracking Analysis (NTA) using a NanoSight LM10 (Malvern). EVs were diluted 40X in 1X PBS prior to measurement. RNA was isolated from EVs by addition of 700 µl of QIAzol reagent (Qiagen) directly into Optiseal tubes following differential centrifugation prior to resuspending the EV pellet. RNA was then purified using the miRNeasy mini kit (Qiagen #217004) according to the manufacturer’s instructions. For mRNA and lncRNA isolation, RNeasy Mini kits (Qiagen #74104) were used. RNA was quantified using a nanospectrophotometer and RNA integrity was assessed by running samples in 15% TBE-Urea gels.

Total protein present in EV samples was determined using the BCA assay (Pierce #23225). Proteins present in EVs were tested by immunoblotting as described previously^21^. Alix, CD9 and GAPDH antibodies were purchased from Cell Signaling; TSG101 (sc-7964) and CD63 (sc-5275) antibodies were purchased from Santa Cruz Biotechnology. Odyssey blocking buffer from LI-COR (catalog # 927-40000) was used for blocking and incubation. Primary and secondary antibodies were diluted 1:1,000 and 1:10,000 in 0.5X blocking buffer. Densitometry was performed using Licor Odyssey software.

### Vascularization bioactivity

To assess vascularization bioactivity *in vitro*, an endothelial gap closure assay was used. 30,000 HUVECs were plated in the wells of gelatin-coated 48-well plates. After 24 hours of incubation in complete EGM2, a scratch was made in each of the wells using the tip of a 200 µl micropipette. Medium was aspirated and the cells were then washed first with 300 µl of 1X PBS and then with 300 µl of EBM2 medium. As positive and negative controls, EGM2 (complete medium) and EBM2 (basal medium) were used. Cells were treated with EVs to make the final concentration of EVs 100 µg/ml based on EV protein quantification. Images were taken of the scratches after 0, 9 and 15 h, and the denuded areas were quantified using ImageJ software. Data reported are for the 15 h time point.

To assess bioactivity *in vivo, a* Matrigel plug assay was performed as described previously^29^. Briefly, 0.4 mL Matrigel (Corning #356231) with or without 100 µg of EVs (in 100 µl PBS) from HUVECs cultured in the presence or absence of 100 mM ethanol was injected subcutaneously into the ventral area of C57Bl/6 mice. After 10 days, mice were euthanized and the Matrigel plugs were excised and fixed in 10% phosphate-buffered formalin before embedding in paraffin and sectioning for histological (hematoxylin and eosin (H&E)) and immunohistochemical analysis. All animal experiments were carried out following protocols approved by the University of Maryland IACUC.

### Immunohistochemical analysis

Sections were affixed to glass slides and deparaffinized in xylene, dehydrated in graded alcohol, and finally hydrated in water. Antigen retrieval was performed by boiling the slides in TE buffer (pH 9.0) for 30 min. CD31 primary antibody (Abcam # ab8364) and goat anti-rabbit IgG H&L secondary antibody (Abcam # ab6719) were used in 1:100 and 1:200 dilutions respectively. After incubation with the primary antibodies at 4 °C overnight, the slides were incubated with peroxidase-conjugated anti-rabbit IgG and stained with diaminobenzidine (DAB) chromogen solution (SK-4105) (both from Vector Laboratories), and then counterstained with hematoxylin. Images were taken with an Olympus BX51 microscope. The percentage of cells with CD31+ was determined by counting the total number of cells and CD31+ cells from each gel section.

### RNA profiling and analysis

Human miRNome miScript miRNA arrays (V16.0, 384-well, SA Biosciences, # MIHS-3216ZE) were used to identify the level of miRNAs in EVs from HUVECs cultured in the presence or absence of 100 mM ethanol. EV RNA was isolated by using miRNeasy mini kits (Qiagen #217004) and cDNA was prepared using miScript II RT kits (Qiagen #21860). As recommended in the protocol, ~350 ng of RNA sample was used to make cDNA for one 384-well plate. The miScript miRNA PCR array reaction volume was kept to 10 μl per well for 384-well plate. PCR was performed using an Applied Biosystems Real Time PCR instrument (ABI 7900 Fast HT) according to manufacturer’s instructions. Finally, data were analyzed using SA Biosciences software: (http://sabiosciences.com/mirnaArrayDataAnalysis.php).

Profiling of mRNAs associated with angiogenesis was carried out in recipient HUVECs exposed to EVs isolated from producer HUVECs cultured in the presence or absence of 100 mM ethanol for 24 h. Quantitative PCR using an ABI 7900 Fast HT machine and employing SsoAdvanced Universal SYBR Green Supermix (Bio-Rad) was used to detect RNA levels in the context of an Angiogenesis PCR array kit (Bio-Rad H384), with settings as recommended for the Supermix reagent. Data were analyzed using the ΔΔCt method. β-actin mRNA and TUG1 lncRNA were selected as controls that were not expected to be affected in control vs. ethanol samples. The sequences of qPCR primers used for this study are available by request.

### miRNA activity assay

To evaluate miRNA targeting of CD34, a pMirTarget reporter plasmid (Origene, catalog# SC213155) containing the coding sequence for the 3′-UTR of CD34 downstream of firefly luciferase was used. The plasmid was transfected into HUVECs that were plated in 12-well plates 24 h prior to transfection using Lipofectamine 2000 (Invitrogen, Carlsbad, CA, USA). After an additional 24 h period, transfected HUVECs were exposed to control solution (PBS) or EVs (100 μg/ml final concentration) from producer HUVECs cultured in the presence of absence of 100 mM ethanol. Cells were harvested after 48 h and lysate was prepared using Luc-Pair™ Duo-Luciferase Assay Kit 2.0 (Genecopoeia#LPFR-P010) according to the manufacturer’s instructions and normalized luciferase levels were determined using a SpectraMax Gemini plate reader.

### Protein detection

Receptor tyrosine kinase (RTK) phosphorylation was investigated using Proteome Profiler Human Phospho-RTK Arrays (R&D Systems #ARY001B). HUVECs were incubated with 100 µg/ml EVs isolated from HUVECs cultured in the presence or absence of 100 mM ethanol for 30 min and a total protein lysate was prepared and applied to antibody pre-coated membranes according to the manufacturer’s instructions. The expression levels of pIR and pEGFR were assessed by standard immunoblotting using the following antibodies: pIR (Tyr 1162/1163) (Santa Cruz #sc-25103 P); pEGFR (Tyr1068) (Cell Signaling #2234 S).

### Transfections

Transfection of HUVECs was performed when cells reached ~50% confluence using HiPerFect reagent (Qiagen #301704). The antagomir of miR-106b-5p (5′-AUC UGC ACU GUC AGC ACU UUA-3′) is a single RNA sequence exactly complementary to miR-106b-5p sequence and was purchased from Integrated DNA Technologies. The concentration of the miR-106b-5p antagomir and negative control was 10 nM. HUVECs mixed with only HiPerFect (mock transfected) and HUVECs transfected with scrambled RNA with HiPerFect were used as controls. After 48 h, cells were harvested and total RNA was prepared to quantify miR-106b-5p level. To measure miR106b-5p levels in EVs, media was collected after 48 h of transfection from T75 flask. The level of miR106b-5p was measured by q-PCR by using primers specific for miR106b-5p. After confirmation of miR106b-5p knockdown in both cells and EVs, a large-scale cell culture was made in T75 flasks and antagomir or scrambled oligo transfections were performed.

A similar approach was employed to knockdown HOTAIR and MALAT1 lncRNAs in HUVECs. TriFECTa RNAi kits against both lncRNAs were purchased from Integrated DNA Technologies (IDT). A pool of three siRNAs or control siRNA was transfected by HiPerFect reagent and the final concentrations of transfected RNA oligos were 50 nM. The knockdown of HOTAIR or MALAT1 lncRNAs from cells and EVs was confirmed by q-PCR using primers specific for each lncRNA.

### Statistical analysis

Parametric statistical tests (one-way analysis of variance (ANOVA) with Bonferroni post-hoc test, 2-sample t-test) were used as appropriate and statistical significance level is indicated for each figure where it was calculated. Data were plotted as mean +/− standard deviation.

### Data availability

All data generated or analyzed during this study are included in this published article (and its Supplementary Information files).

## Electronic supplementary material

Supplementary Information

Supplementary Dataset Table S1

Supplementary Dataset Table S3
