# Supplementary material for: Ethanol Induces Enhanced Vascularization Bioactivity of Endothelial Cell-Derived Extracellular Vesicles via Regulation of MicroRNAs and Long Non-Coding RNAs
Source: Sci Rep. 2017 Oct 23;7:13794. doi: 10.1038/s41598-017-14356-2 (PMC5653762; doi:10.1038/s41598-017-14356-2)
Supplement: Supplementary file 1 — Supplementary Information [file 41598_2017_14356_MOESM1_ESM.pdf]

Ethanol Induces Enhanced Vascularization Bioactivity of Endothelial Cell-Derived Extracellular Vesicles via Regulation of MicroRNAs and Long Non-Coding RNAs

Tek N. Lamichhane, Christopher A. Leung, Lampouguin Y. Douti and Steven M. Jay

Supplementary Information

Supplementary Figures S1-S4

Supplementary Table S2

Figure S1

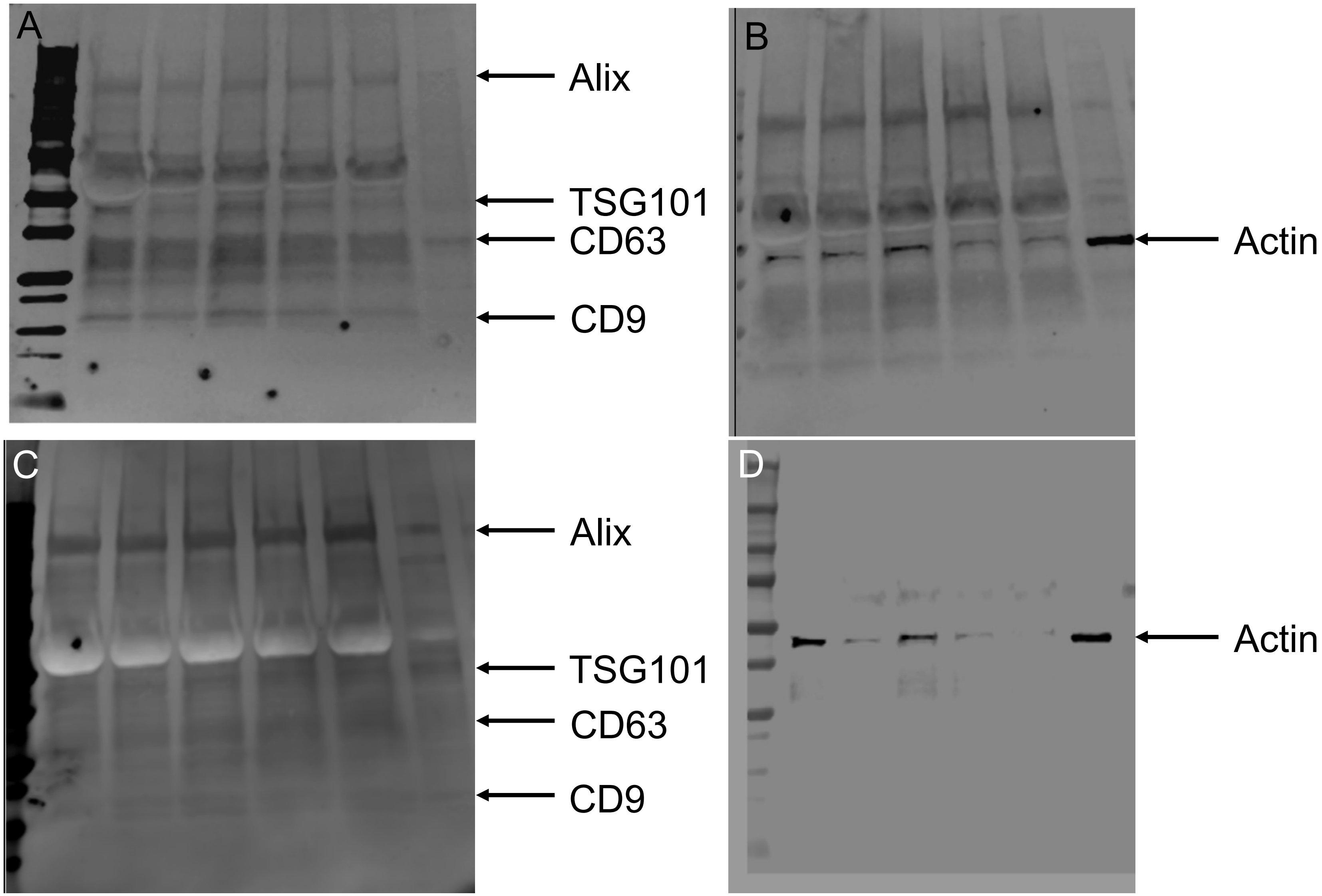

Supplementary Figure S1: Representative blots used in Figure 1C (A, B) and Figure 1D (C, D).

Figure S2

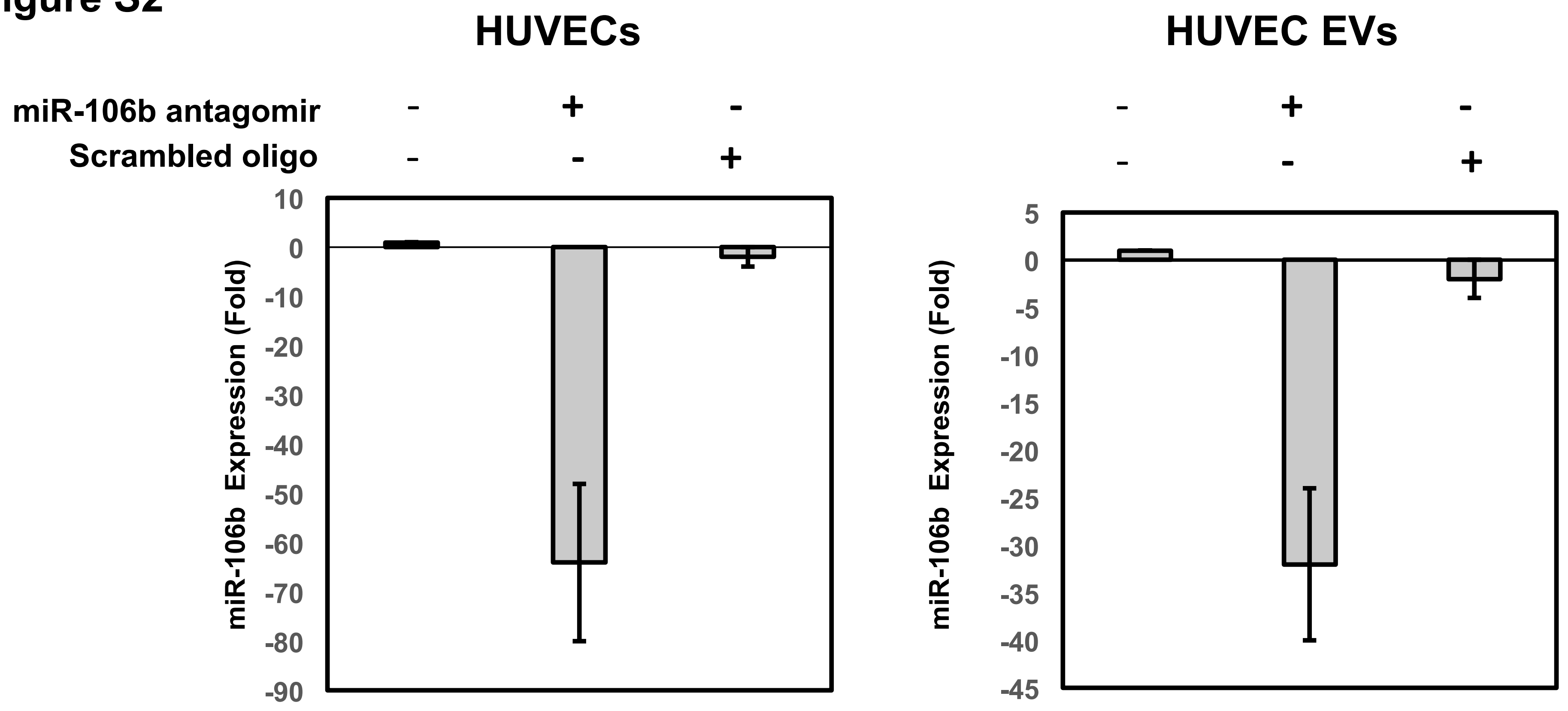

Supplementary Figure S2: The expression levels of miR-106b in HUVECs and HUVEC EVs following transfection of HUVECs by a miR-106b antagomir or a scrambled oligo sequence were determined by qPCR (n=3). Data were normalized to the endogenous level of miR-106b (mock transfected, shown as 1 fold).

Figure S3

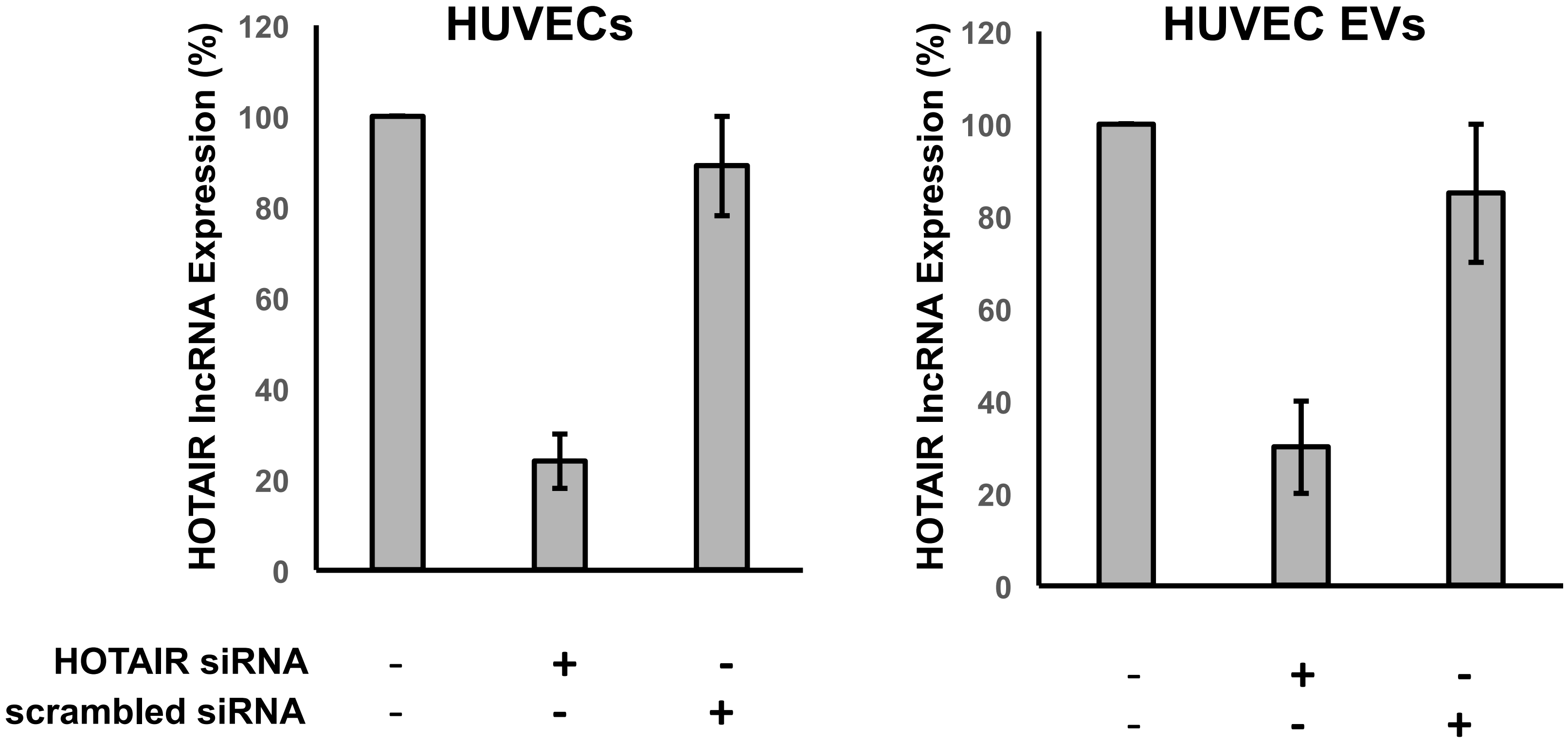

Supplementary Figure S3: The expression levels of HOTAIR lncRNA in HUVECs and HUVEC EVs following transfection of HUVECs by siRNA specific to HOTAIR or a scrambled siRNA sequence were determined by qPCR (n=3). Data were normalized to the endogenous level of HOTAIR lncRNA (mock transfected, shown as 100%) and to TUG1 lncRNA levels.

Figure S4

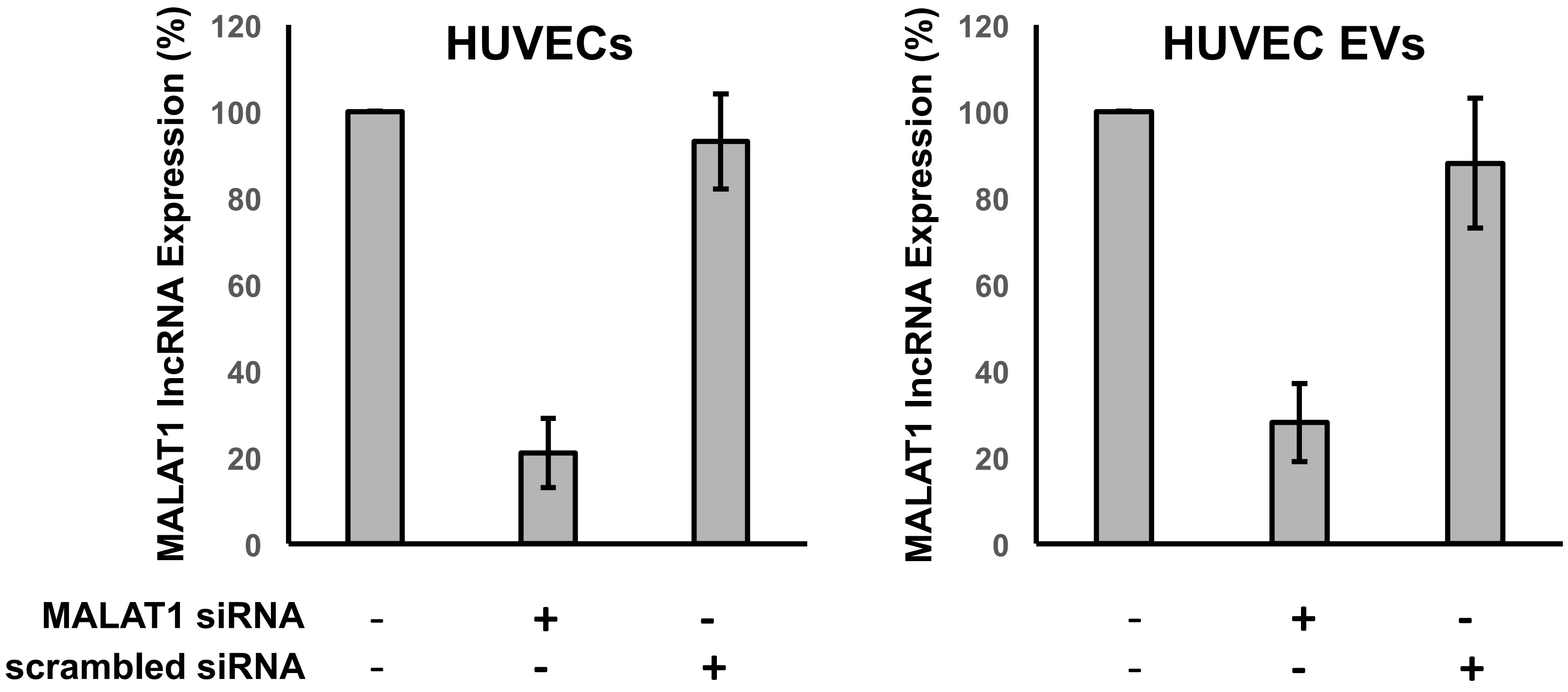

Supplementary Figure S4: The expression levels of MALAT1 lncRNA in HUVECs and HUVEC EVs following transfection of HUVECs by siRNA specific to MALAT1 or a scrambled siRNA sequence were determined by qPCR (n=3). Data were normalized to the endogenous level of MALAT1 lncRNA (mock transfected, shown as 100%) and to TUG1 lncRNA levels.

Supplementary Table S2

List of receptor tyrosine kinases evaluated in Figure 4 (as accessed from the website below (8/23/17))
https://www.rndsystems.com/products/proteome-profiler-human-phospho-rtk-array-kit_ary001b

ALK/CD246
Axl
DDR1
DDR2
Dtk
EGF R
EphA1
EphA2
EphA3
EphA4
EphA5
EphA6
EphA7
EphA10
EphB1
EphB2
EphB3
EphB4
EphB6
ErbB2
ErbB3
ErbB4
FGF R1
FGF R2 alpha
FGF R3
FGF R4
Flt-3/Flk-2
HGF R/c-MET
IGF-I R
Insulin R/CD220
M-CSF R
Mer
MSP R/Ron
MuSK
PDGF R alpha
PDGF R beta
c-Ret
ROR1
ROR2
Ryk
SCF R/c-kit
Tie-1
Tie-2
TrkA
TrkB
TrkC
VEGF R1/Flt-1
VEGF R2/KDR
VEGF R3/Flt-4
